# Adenoviral-mediated correction of methylmalonyl-CoA mutase deficiency in murine fibroblasts and human hepatocytes

**DOI:** 10.1186/1471-2350-8-24

**Published:** 2007-04-30

**Authors:** Randy J Chandler, Matthew S Tsai, Kenneth Dorko, Jennifer Sloan, Mark Korson, Richard Freeman, Stephen Strom, Charles P Venditti

**Affiliations:** 1 National Human Genome Research Institute, National Institutes of Health, Bethesda, MD 20892, USA; 2 Department of Biochemistry and Molecular Biology, Georgetown University, Washington, DC 20057, USA; 3 Miller School of Medicine, University of Miami, Miami, FL 33136, USA; 4 Department of Pathology, University of Pittsburgh School of Medicine, Pittsburgh, PA 15261, USA; 5 Division of Metabolism, Tufts University School of Medicine, Boston, MA 02111, USA; 6 Division of Transplantation, Tufts University School of Medicine, Boston, MA 02111, USA

## Abstract

**Background:**

Methylmalonic acidemia (MMA), a common organic aciduria, is caused by deficiency of the mitochondrial localized, 5'deoxyadenosylcobalamin dependent enzyme, methylmalonyl-CoA mutase (MUT). Liver transplantation in the absence of gross hepatic dysfunction provides supportive therapy and metabolic stability in severely affected patients, which invites the concept of using cell and gene delivery as future treatments for this condition.

**Methods:**

To assess the effectiveness of gene delivery to restore the defective metabolism in this disorder, adenoviral correction experiments were performed using murine *Mut *embryonic fibroblasts and primary human methylmalonyl-CoA mutase deficient hepatocytes derived from a patient who harbored two early truncating mutations, E224X and R228X, in the *MUT *gene. Enzymatic and expression studies were used to assess the extent of functional correction.

**Results:**

Primary hepatocytes, isolated from the native liver after removal subsequent to a combined liver-kidney transplantation procedure, or *Mut *murine fibroblasts were infected with a second generation recombinant adenoviral vector that expressed the murine methylmalonyl-CoA mutase as well as eGFP from distinct promoters. After transduction, [1-^14^C] propionate macromolecular incorporation studies and Western analysis demonstrated complete correction of the enzymatic defect in both cell types. Viral reconstitution of enzymatic expression in the human methylmalonyl-CoA mutase deficient hepatocytes exceeded that seen in fibroblasts or control hepatocytes.

**Conclusion:**

These experiments provide proof of principle for viral correction in methylmalonic acidemia and suggest that hepatocyte-directed gene delivery will be an effective therapeutic treatment strategy in both murine models and in human patients. Primary hepatocytes from a liver that was unsuitable for transplantation provided an important resource for these studies.

## Background

Methylmalonic acidemia (MMA) is a common organic aciduria characterized by elevated levels of methylmalonic acid in the fluids and tissues of the body [1]. Affected patients have a well-recognized clinical phenotype [2,3], characterized by acute metabolic decompensation, and a guarded long-term prognosis [4-7]. The metabolic disorder commonly results from mutations in the methylmalonyl-CoA mutase (*MUT*) gene [8,9]. This enzyme catalyzes the formation of succinyl-CoA from L-methylmalonyl-CoA, a critical intermediate step in the conversion of propionyl-CoA to succinyl-CoA. Two enzymatic phenotypes of *mut *methylmalonic acidemia are recognized. Fibroblasts from *mut*^*o *^patients have no detectable or residual enzyme activity in their fibroblasts [10] while those from *mut*^- ^patients have enzyme activity that is markedly reduced but typically cobalamin-responsive *in vitro *[11,12].

The precise etiology of the complications seen in methylmalonic acidemia are uncertain, and even patients under treatment are at risk for intermittent metabolic decompensation, pancreatitis[13], infarction of the basal ganglia[14,15], and renal failure[7,16]. The main treatment of vitamin B-12 non-responsive methylmalonic acidemia includes nutritional management and alkali replacement [2,3] as well as carnitine supplementation[17]. Despite restriction of dietary precursors, many patients exhibit metabolic fragility that can be life-threatening. Some individuals have undergone liver and combined liver-kidney transplantion to eliminate the metabolic instability that is characteristic of the condition [18-21]. Liver and liver-kidney transplant recipients do not experience life-threatening ketoacidotic attacks, but remain at risk for renal disease [20] and infarction of the basal ganglia[21]. While the timing, indications, efficacy and outcomes of patients undergoing these procedures have not been fully defined, the metabolic stability conferred after liver transplantation indicates that the liver plays a critical role in methylmalonyl-CoA metabolism. This suggests that hepatocyte-directed viral or cell therapies may provide viable alternatives to liver transplantation in MMA patients who cannot find a donor organ or for whom the risk of transplantation is prohibitive. Hepatocyte transplantation has been used clinically and has been shown to provide partial or complete correction of several liver based metabolic diseases [22] and may represent a viable alternative to transplantation in affected MMA patients.

Previous attempts to use viral mediated gene delivery to correct the metabolic defect in *mut*^*o *^primary fibroblasts were inefficient and required multiple cycles of retroviral infection and selection to restore propionate flux[23]. In this report, we describe the generation of a versatile adenoviral vector that co-expresses the methylmalonyl-CoA mutase gene and an eGFP reporter from independent promoters. The virus efficiently restored propionate metabolism in methylmalonyl-CoA mutase deficient murine fibroblasts as well as human hepatocytes derived from a patient with *mut*^0 ^methylmalonic acidemia and was easily tracked by *in vivo *fluorescence after direct intraheptic injection in mice. The results demonstrate the efficacy of viral correction of the enzymatic defect in varied cell types from mouse and man and provide evidence for viral based gene delivery approaches to treat methylmalonic acidemia. The extent of correction achieved in primary *mut*^0 ^hepatocytes was greater than that seen in fibroblasts or control hepatocytes and suggests that hepatocyte-directed gene delivery will be an effective therapeutic strategy in both murine models and in human patients.

## Methods

### Patient medical history

Human hepatocytes used in viral correction experiments were derived from the discarded liver of a 5 year old boy with *mut*^*o *^class methylmalonic acidemia undergoing a combined renal and hepatic transplant procedure. The patient initially presented with hyperammonemia and metabolic crisis in the presence of extreme methylmalonic acid elevations on the second day of life. Complementation and [1-^14^C] propionate incorporation studies on skin fibroblasts indicated a *mut*^0 ^lesion. Subsequent sequencing of the *MUT *gene revealed two early nonsense mutations, E224X and R228X[9]. At the age of five, the patient underwent a combined liver kidney transplant from a deceased donor in which the whole organ liver and left kidney allografts were implanted in one procedure.

### Cell lines

*Mut *and wild-type murine embryonic fibroblasts have been described[24,25] and were isolated after a timed mating between mice carrying a targeted deletion of the *Mut *gene.

Hepatocytes from the affected patient were isolated after the liver was removed as part of elective combined liver-kidney transplantation. The liver was considered a discarded surgical specimen because it was not suitable for transplantation and was donated by the family for research use. Patient studies were conducted in compliance with the Helsinki Declaration and were approved by the National Human Genome Research Institute Institutional Review Board as part of NIH study 04-HG-0127 "Clinical and Basic Investigations of Methylmalonic Acidemia and Related Disorders" after informed consent was obtained. Hepatocyte isolation from resected liver specimens was approved by the University of Pittsburg Institutional Review Board (IRB Number 0411142). Immediately upon surgical removal of the native liver the organ was flushed with ice-cold University of Wisconsin solution and shipped on wet ice to the University of Pittsburgh cell isolation facility established as part of the NIH-funded Liver Tissue Procurement and Distribution System. Hepatocytes were isolated from as described by Strom et al. [26,27], with some minor changes described here. Briefly, cells were isolated from the entire liver by a 3-step collagenase perfusion protocol. Catheters were sewn in place into the 3 of the large hepatic veins and the liver was placed in a sterile plastic bag, and connected to a pump that delivered perfusate at approximately 80 mls/minute/catheter. The liver was sequentially perfused with 1 liter of calcium and magnesium-free HBSS (Cambrex, Walkersville, MD) containing EGTA (1 mM), 1 liter of HBSS without EGTA. Finally, 1 liter of Minimum Essential Medium Eagle (EMEM, Cambrex, Walkersville, MD) containing 250 mg collagenase (Type XI, Sigma, St. Louis, MO) and 50 mg of DNAase (Sigma, St. Louis, MO) was recirculated until the tissue was digested (approximately 29 minutes). Following digestion, tissue was placed in a sterile beaker, covered in ice-cold buffer EMEM and thoroughly chopped with a sterile scissors. Buffer and cells were decanted through sterile gauze covered funnels. Hepatocytes were enriched relative to nonparenchymal cells by 3 consecutive centrifugation steps at 75 × g for 5 minutes each. The final cell pellet was resuspended in hepatocyte maintenance medium (HMM, Cambrex, Walkersville, MD) and the viability was assessed by trypan blue exclusion. Visual inspection revealed greater than 95% parenchymal hepatocytes that were then plated on six well plates as described [26]and allowed to attach for 4 hr, washed twice in serum-free media to remove dead and unattached cells and maintained thereafter by daily changes with serum-free HMM media. Control hepatocytes were isolated from a second donor, a 63 year old Male, not suspected to have primary liver disease or methylmalonic acidemia.

### Adenoviral vector construction and production

A murine methylmalonyl-CoA mutase cDNA that contained a consensus Kozak sequence and minimal untranslated regions was isolated from C57/BL6 liver RNA after RT-PCR, sequenced and tested for enzymatic activity after expression in yeast using the succinate-thiokinase linked assay[25]. The gene was then cloned as an EcoRI fragment into the polylinker of pShuttle between the CMV promoter and the Sv40 polyadenylation signal (ViraQuest Inc., North Liberty, IA). This E1 shuttle expressing Mut from the CMV promoter was then used in the RAPAd^® ^(U.S. Patent #6,830,920) adenovirus construction system with an RSV eGFP expressing E3 backbone[28]. The dual-expressing virus was derived by recombination after co-transfection of the E1 shuttle and backbone into HEK293 cells. 7–10 days after the appearance of viral foci, the plates were harvested and amplified, and then virus particles were isolated by two rounds of CsCl gradients and dialyzed against storage buffer (ViraQuest Inc., North Liberty, IA). Vector genomes and plaque forming units (PFU) were measured in the final adenoviral preparations. Animal studies were reviewed and approved by the National Human Genome Research Institute Animal User Committee. The cell culture correction experiments used virus at a multiplicity of infection (MOI) of 1000. Direct intrahepatic injections of 1.0 × 10^10 ^particles (3.3 × 10^8 ^PFU) of the adenovirus in mixed background (C57Bl6xSv129Ev) neonatal mice were accomplished using a 32 gauge Hamilton syringe. The animals were sacrificed 4 days later for dissection and analysis of eGFP expression.

### Incorporation studies

Methylmalonyl-CoA mutase activity was determined by measuring [1-^14^C] propionate incorporation into macromolecules as described[29]. All propionate incorporation assays were performed in triplicate. [1-^14^C] Sodium propionate was purchased (Perkin Elmer, Boston, MA) as a custom preparation at a specific activity of 55.0 mCi/mmol (2 mCi/ml).

### Western blotting

Whole cell extracts from the cell lines were analyzed by immunoblotting and probed with affinity purified, rabbit polyclonal antisera raised against the murine methylmalonyl-CoA mutase enzyme or beta-actin (Abcam, Cambridge, MA). The anti-mutase antibody was used at a dilution of 1:750, and beta-actin was used at 1:5,000. Goat anti-rabbit (Chemicon, Temecula, CA) was used as a secondary antibody at a dilution of 1:10,000 with chemiluminescent detection (Pierce Biotechnology, Rockford, IL). Recombinant murine methylmalonyl-CoA mutase enzyme was used as a positive control in Western blot analysis experiments. Human liver extracts were prepared from an anonymous liver donor not known to have methylmalonic acidemia or the *mut*^0 ^patient described above.

## Results

### Bifunctional adenoviral construction

**Figure 1:**
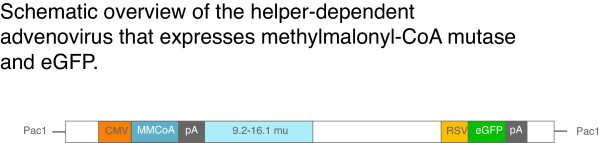
Schematic of adenovirus. An overview of the recombination strategy used to construct the E1, E3 replaced adenovirus. CMV – cytomegalovirus promoter. MMCoA – murine methylmalonylCoA mutase. 9.2–16.1 mu – region of homologous recombination. RSV – Rous sarcoma virus promoter. eGFP – green fluorescent protein. PA – Sv40 polyadenylation signal.

Transfection and recombination in HEK293 cells using the RAPAd^® ^adenovirus method was used to produce replication deficient adenovirus particles that express the murine methylmalonyl-CoA mutase gene in the E1 region and eGFP from the E3 region[28]. The resulting replication deficient adenovirus expresses both genes from two independent promoters, Mut from the CMV and eGFP from the RSV (Figure 1). The virus was expanded, amplified and concentrated to an infectious titer of 1.2 × 10^12 ^particles/ml (corresponding to 4.0 × 10^10 ^PFU/ml), indicating its stability during growth and replication. The strong viral promoters were selected to promote high-level expression in a wide spectrum of tissues types, *in vivo *and *in vitro*.

### Reporter expression in the murine liver

**Figure 2:**
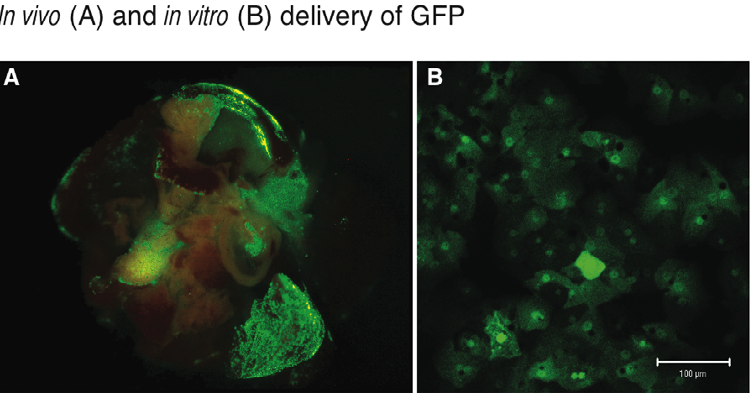
*In vivo *delivery of GFP to the murine liver and *in vitro *expression in primary *mut*^*o *^human hepatocytes. A. A wild-type neonatal mouse was treated on the first day of life by direct intrahepatic injection. The liver was harvested on the fourth day. The green-areas represent tissue that expresses eGFP. B. eGFP expression in primary *mut*^*o *^human hepatocytes 24 hours after infection with the bi-functional adenovirus.

The bifunctional adenovirus was delivered by direct hepatic injection into the liver of one day old wild-type mice (N = 8). A total of 1.2 × 10^10 ^(3.3 × 10^8 ^PFU) adenoviral particles were administered in a total volume of 10 microliters. Four days post injection the animals were sacrificed and the organs were inspected for fluorescence using a dissecting microscope equipped with a GFP filter. The GFP expression was easily visualized, with a higher concentration of signal seen in the lobe that was likely the direct target of injection (Figure 2A). The presence of green fluorescence, not observed in the uninjected mice, indicates that the RSV-eGFP promoter functioned *in vivo*. Other organs, such as the heart and kidney, lacked signal and GFP expression was not observed in the uninjected mice. None of the wild type mice injected at this dose perished, suggesting that the virus and procedure were well tolerated.

### Adenoviral correction of methylmalonyl-CoA mutase deficient murine embryonic fibroblasts (MEFs)

**Figure 3:**
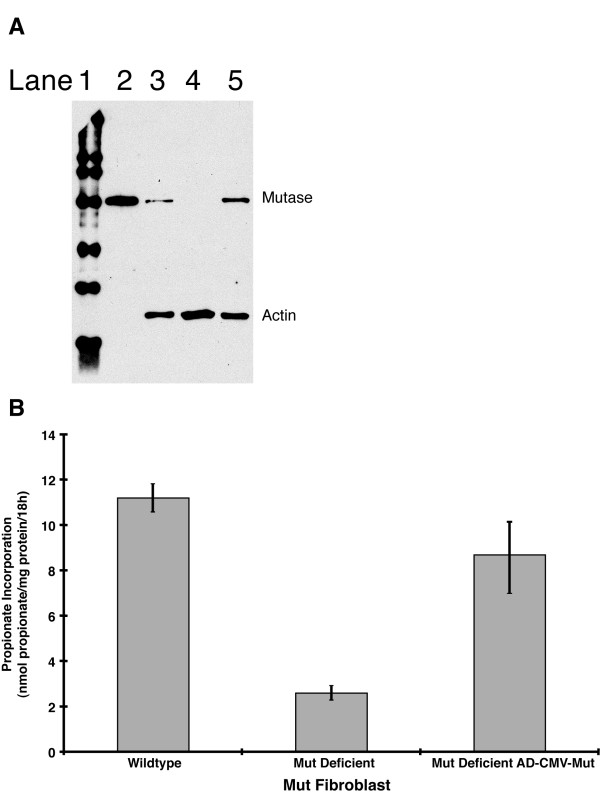
Expression and functional studies of methylmalonyl-CoA mutase in *Mut *murine embryonic fibroblasts. A. Western analysis of protein lysates from MEFs probed with methylmalonyl-CoA mutase antibody (78 kDa, labeled mutase) and cross-reactive beta-actin loading control (47 kDa, labeled actin). All wells had 10 micrograms of total protein loaded. Lane 1: Marker; Lane 2: Recombinant murine methylmalonyl-CoA mutase; Lane 3: Wild-type MEF; Lane 4: Murine methylmalonyl-CoA mutase knock-out MEFs; Lane 5: Adenoviral-corrected murine methylmalonyl-CoA mutase knock-out MEFs. B. Murine methylmalonyl-CoA mutase knock-out murine embryonic fibroblasts (uncorrected and corrected with adenovirus) were assayed for [1-^14^C] propionate incorporation over 18 hours. Activity was normalized to total protein content of the extracts. The samples were analyzed in triplicate. The bars around the average represent +/- one standard deviation.

*Mut *MEFs derived from methylmalonyl-CoA mutase knock-out mice were used to determine the efficacy of gene correction from the virus[24]. Methylmalonyl-CoA mutase protein and mRNA are not detectable in these cells (Figure 3 Lane 4; Chandler et al, submitted). The virus was incubated at a multiplicity of infection of 1000 for 16 hours. The cells were washed and then used to determine the extent of correction by Western blotting 72 hours post transduction. In parallel, enzymatic function was measured using macromolecular [1-^14^C] propionate incorporation. A wild-type murine fibroblast cell line served as a positive control in these experiments. Previous studies on the same cells using an E1 replaced, eGFP expressing adenovirus at the same MOI showed no effect on [1-^14^C] propionate incorporation and no gross cellular toxicity (Chandler et al, data not presented).

After correction, expression at the protein level was completely restored in the knock-out MEFs that had been infected. Figure 3A (Lane 4) shows that the *Mut *cells make no detectable methylmalonyl-CoA mutase protein compared to the wild type control (Lane 3), which has a single band migrating at 78 kDa. This band corresponds to the expected size of the murine enzyme after processing and is the same size as recombinant murine methylmalonyl-CoA mutase (Figure 3A, Lane 2). When normalized to actin, the enzymatic expression level in infected cells appears increased over wild type cells (compare Figure 3A Lane 5 to Lane 3).

We also measured the activity of the methylmalonyl-CoA mutase enzyme by evaluating propionate metabolism in *Mut *fibroblasts and compared it to *Mut *MEFs exposed to the virus. As demonstrated in Figure 3B, the [1-^14^C] propionate incorporation in the transduced cells nearly corrects to the levels observed in wild type cells, demonstrating that viral directed expression provides functional enzymatic correction.

### Adenoviral correction of methylmalonyl-CoA mutase deficient primary hepatocyte cell line

The native liver from a patient with *mut*^*o *^methylmalonic acidemia was harvested as part of a combined liver-kidney transplantation. Hepatocytes were isolated using collagenase perfusion. Cells were plated at a density of 5 × 10^5 ^per well on collagen-coated 6-well plates (Falcon) and appeared large, hexagonal and healthy. Control hepatocytes, derived from an 63 year old anonymous male donor, were prepared in an identical fashion and were used to measure native [1-^14^C] propionate in an independent experiment.

**Figure 4:**
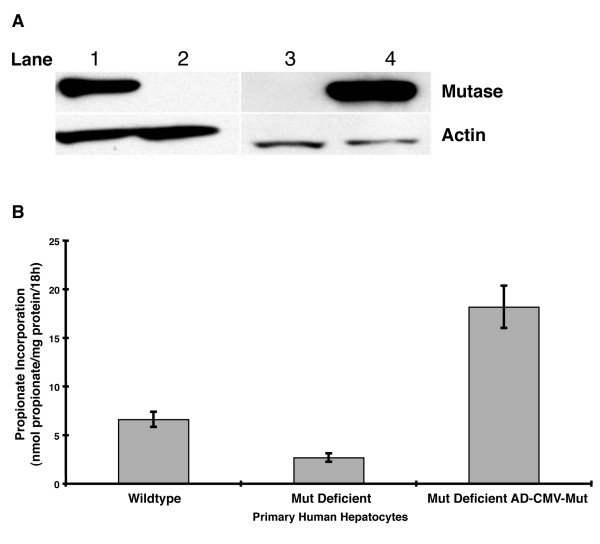
Expression and functional studies of methylmalonyl-CoA mutase in primary *mut*^*o *^human hepatocytes. A. Western analysis of protein lysates from primary human hepatocytes probed with methylmalonyl-CoA mutase antibody (78 kDa, labeled mutase) and cross-reactive beta-actin loading control (47 kDa, labeled actin). Lane 1: human control whole liver extract (20 micrograms) Lane 2: *mut*^*o *^whole liver extract (20 micrograms); Lane 3: human primary *mut*^*o *^primary human hepatocytes (10 micrograms); Lane 4: adenoviral corrected *mut*^*o *^primary human hepatocytes (10 micrograms). B. Human hepatocytes (wild type, *mut*^*o *^and *mut*^*o *^corrected with adenovirus) were assayed for [1-^14^C]-propionate incorporation over 18 hours after viral incubation for 48 hrs. Activity was normalized to total protein content of the extracts. The samples were analyzed in triplicate. The bars around the average represent +/- one standard deviation.

The hepatocytes were infected at a MOI of 1000, as previously described. Twenty-four hours after infection the cells exhibited uniform GFP expression (Figure 2B). To access viral mediated correction, functional studies were performed on the hepatocytes with and without exposure to the adenovirus for 48 h. Compared to a control human liver extract (Figure 4A, lane 1), extracts derived from the human *mut*^*o *^liver (Figure 4A, lane 2) and uninfected hepatocytes (Figure 4A, lane 3) revealed no cross reactive material even after prolonged exposure of the Western blot. Genetic studies on this patient previously identified two nonsense mutations (E224X and R228X)[9] and these experiments confirmed that there was no read through. Post-infection, 10 micrograms of the *mut*^*o *^corrected hepatocyte extract exhibited a large increase in immunoreactive enzyme as assessed by Western blotting (Figure 4A, Lane 4). When compared to the control human liver extract (Figure 4A, Lane 1), which contained 20 micrograms of protein, the corrected cells (Figure 4A, Lane 4) have a substantially larger band, suggesting an increased capacity in the virally infected cells for expression of the methylmalonyl-CoA mutase enzyme, even if normalization to actin is not considered in the comparison.

Enzymatic studies revealed a greatly increased metabolic capacity of the mutant hepatocytes after viral correction (Figure 4B). [1-^14^C] propionate incorporation in the virus exposed mut^0 ^hepatocytes increased to approximately 20 nmol/mg/18 h (a factor of 7) over the uninfected cell line and exceeded the activity of control primary hepatocytes (Figure 4B). It should be noted that the control hepatocytes were derived from an older patient and hence may have a reduced propionate capacity compared with what might be observed in hepatocytes from a child, because of issues such as growth rate and/or other age related phenomenon. Unfortunately, an age and sex matched liver was not available for similar studies. The greatly increased [1-^14^C] propionate incorporation displayed by the *mut*^*o *^cell line after viral correction indicates that hepatocytes have a large capacity for propionate metabolism and is consistent with the robust expression observed in the Western studies. In all the adenoviral delivery experiments, the infected cells appeared morphologically normal at the time enzymatic and Western studies were performed.

## Discussion

In these studies, we developed and tested adenoviral gene delivery in murine *Mut *fibroblasts and human *mut*^*o *^hepatocytes. The virus allowed the expression of a linked but distinctly transcribed *cis *eGFP minigene for rapid visual tracking of infectivity. After direct hepatic injection and incubation of primary human hepatocytes and MEFs (data not presented) with the virus, green fluorescence was readily apparent, demonstrating that the reporter functioned well *in vivo *and *in vitro*. Highly concentrated and purified virus allowed for small delivery volumes *in vivo *without adverse effects in the doses administered and was highly active in murine and human cell culture systems.

Murine, as opposed to human, *Mut *cDNA was selected for viral correction experiments because previous studies demonstrated the ability of the murine gene to correct human *mut*^*o *^fibroblasts[30], despite the divergent sequence of its mitochondrial importation signal[31]. This is in contrast to other cross-species correction studies that have demonstrated the need for a species-specific mitochondrial importation signal[32]. Additionally, proof of principle studies using the mouse gene to correct murine mutant cells are needed before gene therapy experiments proceed in animal models of methymalonic acidemia. Retroviral delivery of the methylmalonyl-CoA mutase gene followed by selection has been used to correct a mutant methylmalonyl-CoA mutase deficient cell line but required multiple rounds of viral superinfection and selection to restore propionate metabolism[23]. Delivery with selection may represent an option suitable for retroviral or transposon mediated correction strategies but the rapidity of symptom onset in the *Mut *mice suggests that correction must be accomplished very early to be effective, possibly even *in utero *[33,34]. Adenoviral vectors can deliver a large dose of bioactive material quickly with highly efficient uptake and expression in the liver. The vector described here does not carry a selectable marker or provide permanent correction. Nevertheless, it may rescue neonatal *Mut *animals so that they can be studied at a later age. In this fashion, the treated mutants can serve as models for investigating the development of late, MMA-related pathophysiology such as basal ganglial infarction, pancreatitis and renal disease and also be used to assess other cell and viral based therapies.

In these studies, adenoviral correction of primary human *mut*^*o *^heptocytes was especially effective. Other authors have demonstrated that hepatocytes possess an increased basal capacity for propionate metabolism[23,35] as our results also suggest. Furthermore, when individual murine tissues were examined for methylmalonyl-CoA mutase activity, the liver possessed a large amount of active enzyme, with an total activity of > 1400 nmol succinate formed/h per mg of protein[31]. Taken together with the observations that *mut *liver transplant recipients are quite stable despite having massive methymalonic acidemia[20,36,37], the concept of targeting the liver for gene delivery appears attractive.

Our results show that adenoviral mediated gene delivery can greatly increase propionate metabolism in mutant hepatocytes. The genetic background of the *Mut *murine MEFs and human *mut*^*o *^hepatocytes is similar because both are null at the protein level as confirmed by Western analysis (Figures 3 and 4). Although many affected *mut*^*o *^patients harbor missense mutations [8,9] and interallelic effects are a well recognized phenomenon[38], the extent of correction we achieved is likely to be influenced only by gene expression from the viral cassette, not partial rescue or stabilization of a mutant protein due to the nature of the nonsense mutations present in the patient.

Liver transplantation has been used to augment medical therapy for both methylmalonic[39] and propionic acidemia[40]. Affected individuals can have mild steatosis and transaminitis but maintain normal synthetic liver function[2]. However, when these patients have undergone hepatic transplantation, the native livers have not been used in a domino fashion, as has been done in other metabolic disorders of branched chain aminoacid oxidation, such as maple syrup urine disease[41]. Most methymalonic acidemia patients undergoing liver transplantation will have the organ harvested and discarded or sent to pathology. We have demonstrated that an otherwise functionless liver can be used for scientific studies that have relevance to hepatocyte therapy and gene delivery and suggest that, in the future, the disposition of harvested organs from methylmalonic acidemia and other patients be carefully considered. Mutant human hepatocytes isolated from otherwise unusable donor livers, particularly those affected by metabolic disorders, might also be used to create hepato-specific genetic mosaic animals by transplantation approaches[42]. This approach would allow the *in vivo *propagation of affected human cells in mice and might permit experiments not possible in either species. Future efforts will focus on adenoviral mediated gene delivery to rescue the neonatal lethal phenotype seen in *Mut *knock-out mice, an approach that now has firm experimental evidence of efficacy.

## Conclusion

Our results demonstrate, for the first time, efficacious viral mediated gene correction in primary murine and human methylmalonyl-CoA mutase deficient cell lines. Primary hepatocytes derived from an affected patients liver that was unsuitable for transplantation provided an important resource for these studies. In the future, hepatocyte-directed gene therapy should provide an effective therapeutic treatment in both murine models and in human patients.

## Abbreviations

MMA (methylmalonic acidemia), MUT (human methylmalonyl-CoA mutase), Mut (murine methylmalonyl-CoA mutase), *mut*^*o *^(vitamin B12 non-responsive methylmalonic acidemia), MEF (murine embryonic fibroblasts), MOI (multiplicity of infection), PFU (plaque forming units), CMV (cytomegalovirus), RSV (Rous sarcoma virus), eGFP (enhanced green fluorescent protein)

## Competing interests

The author(s) declare that they have no competing interests.

## Authors' contributions

RJC performed viral, murine, and biochemical studies and drafted the manuscript; MST performed viral, murine, and biochemical studies; KD isolated hepatocytes; SS provided research materials and aided with manuscript revision; JS provided patient care, clinical information and aided with manuscript revision, MK and RF provided patient care, clinical information and procured samples; and CPV constructed the adenoviral vector and was responsible for the concept of the study, its design and coordination and drafted the manuscript. All authors read an approved the final manuscript.

## Pre-publication history

The pre-publication history for this paper can be accessed here:


